# Short-term melatonin supplementation decreases oxidative stress but does not affect left ventricular structure and function in myxomatous mitral valve degenerative dogs

**DOI:** 10.1186/s12917-021-03125-z

**Published:** 2022-01-07

**Authors:** Wanpitak Pongkan, Chanon Piamsiri, Sirada Dechvongya, Verasak Punyapornwitthaya, Chavalit Boonyapakorn

**Affiliations:** 1 Department of Veterinary Biosciences and Veterinary Public Health, Faculty of Veterinary Medicine, Chiang Mai University, Chiang Mai, 50100 Thailand; 2 Integrative Research Center for Veterinary Circulatory Sciences, Faculty of Veterinary Medicine, Chiang Mai University, Chiang Mai, 50100 Thailand; 3 Veterinary Cardiopulmonary Clinic, Small Animal Hospital, Faculty of Veterinary Medicine, Chiang Mai University, Chiang Mai, 50100 Thailand; 4 Department of Companion Animal and Wildlife Clinic, Faculty of Veterinary Medicine, Chiang Mai University, Chiang Mai, 50100 Thailand; 5 Department of Food Animal Clinic, Faculty of Veterinary Medicine, Chiang Mai University, Chiang Mai, 50100 Thailand

**Keywords:** Dog, Left ventricular structure and function, MDA, Melatonin, Myxomatous mitral valve degeneration

## Abstract

**Background:**

Cardiac wall stress and high oxidative stress are often found in cases of myxomatous mitral valve degenerative (MMVD) disease and can lead to myocardial injuries and cardiac dysfunction. Melatonin, an antioxidant, has been shown to exert cardioprotection in laboratory animal models. However, its effect on metabolic parameters and left ventricular (LV) adaptation in MMVD dogs has rarely been investigated. This clinical trial hypothesized that a melatonin supplement for 4 weeks would improve metabolic parameters, LV structure (diameters and wall thickness), and LV function in MMVD dogs. Blood profiles, echocardiograms, and oxidative stress levels were obtained from 18 dogs with MMVD stage B2 and C at baseline and after prescribed Melatonin (2 mg/kg) for 4 weeks. Eleven dogs with MMVD stage B2 and C, which received a placebo, were evaluated as a control group.

**Results:**

In this clinical trial, the baseline plasma malondialdehyde (MDA) was no different between the treatment and placebo groups. The post-treatment plasma MDA levels (4.50 ± 0.63 mg/mL) in the treatment group was significantly decreased after 4 weeks of melatonin supplementation compared to pre-treatment levels (7.51 ± 1.11 mg/mL) (*P* = 0.038). However, blood profiles and LV structure and function investigated using echocardiography were found not to different between pre-and post-treatment in each group. No adverse effects were observed following melatonin supplementation.

**Conclusions:**

This clinical trial demonstrated that a melatonin supplement for 4 weeks can attenuate oxidative stress levels in MMVD dogs, especially in MMVD stage C, but does not result in LV structural changes or LV function in MMVD dogs of either stage B2 or stage C.

## Background

Heart diseases are a type of disorder which can be observed in most small animal veterinary practices. Myxomatous mitral valve degeneration (MMVD) is the most common cardiovascular disease in dogs, which causes valvular insufficiency and chronic volume overload [1]. The volume overload situation in cardiovascular diseases can result in structural and functional changes at the cellular and whole-organ levels and can lead to heart failure [2]. Heart failure is a multifactorial syndrome described as the cardiac muscle’s structural or functional inability to provide adequate blood flow to meet the metabolic demands of the animal’s body [2, 3]. Alteration of blood flow or blood supply can occur in myocardial cells, vascular cells, and the extracellular matrix [4]. Moreover, prolonged increase in ventricular volume (preload) creates physiological stress on the ventricular wall and resulting in increase more ATP and oxygen consumption [3–5]. At the cellular level, alterations in the cardiac mitochondrial metabolic pathways can result in cardiomyocyte injuries [3–5].

The level of oxidative stress is one of the most crucial factors in animals suffering from heart failure [6, 7]. Many studies in animal models have reported that reactive oxygen species (ROS) and oxidative stress are a critical mediator, inducing myocardial damage such as volume overload-induced heart failure (HF) in dogs [6–8]. The measurement of stable by-products modified under conditions of oxidative stress is used to determine the level of oxidative stress, e.g., plasma MDA, tumor necrosis factor-α (TNF-α), glutathione (GSH), and lipid hydroperoxide [9–11]. Malondialdehyde (MDA) is a lipid peroxidation product of polyunsaturated fatty acids from the phospholipid bilayer in the cell membrane [10]; the MDA-reactive products can be detected by measuring thiobabituric acid levels [12].

Melatonin is an endogenous indoleamine hormone produced by pinealocytes. Production is regulated by the suprachiasmatic nucleus (SCN) located in the anterior part of the hypothalamus [13]. In most diurnal mammals, melatonin is secreted at night and follows a strong circadian rhythm. The maximum plasma melatonin level is highest at 2-4 AM and lowest (almost zero) during the daytime [14, 15]. Its receptor-independent actions demonstrate its multifunctional activity, e.g., potent antioxidant, anti-apoptosis, anti-necrosis, anti-lipid peroxidation and inflammation anti-excitatory, immunomodulatory, vasomotor, and metabolic proprieties [16, 17]. Previous studies in laboratory animal models have also reported melatonin’s benefits in cases of heart failure [18, 19] by the exertion of numerous cardioprotective actions, including increased autophagy and mitophagy, anti-apoptosis, and anti-necrosis [16, 20, 21]. In a human study, the circulating melatonin levels were low in congestive heart failure patients [22]. In addition, previous studies in rats and mice with cardiac hypertrophy (laboratory animal models) have demonstrated that melatonin can decrease cardiac hypertrophy by reducing extracellular matrix deposition [19] and by reducing the fibrosis of cardiac muscle cells [18]. Melatonin thus has beneficial anti-hypertensive properties for both systemic hypertension and pulmonary hypertension [23, 24]. Furthermore, left ventricular ejection fraction (LVEF) improvements have been detected in human patients with heart failure after melatonin treatment [25]. These beneficial cardiovascular effects of melatonin could prevent or reduce pathological remodeling [21, 26]. Many previous in vitro and in vivo animal model studies as well as clinical studies in humans support the benefits of melatonin administration as an adjunctive therapy in cardiovascular diseases [18–21, 23–25, 27]. However, there have been few studies of the use of melatonin in veterinary medicine. The purpose of this study was to investigate the beneficial effects of melatonin on LV structural changes (cavity diameter and wall thickness), LV function, blood profiles, and oxidative stress in dogs with MMVD stage B2 and stage C suffering from cardiac remodeling and volume overload. It was hypothesized that melatonin could induce positive changes in LV structure (cavity diameter and wall thickness), LV function, and attenuate oxidative stress levels in MMVD stage B2 and stage C dogs.

## Results

### Short-term (4 weeks) melatonin supplement did not affect the body condition score or vital signs of MMVD dogs

In this study, the mean ± SD of age in control and treatment group was 12.27 ± 3.0 and 12.39 ± 2.3 year, respectively. There were not statistically different between groups. Additionally, there were not statistically significantly differences in body condition score, body weight, or vital signs (heart rate, pulse rate, SBP, DBP, and MAP) between pre- and post-treatment (4 weeks) in placebo and melatonin administration. This study suggests that a melatonin supplement for 4 weeks does not affect body condition score or vital signs in MMVD dogs (Table 1).

**Table 1:** Body weight, body condition score and vital signs of myxomatous mitral valve degenerative dogs pre- and post-treatment in the control and treatment groups

Parameter	Control group (*n* = 11)		Treatment group (*n* = 18)	
	Pre-treatment	Post-treatment	Pre-treatment	Post-treatment
BW (kg)	5.4 (5.40-6.15)	5.4 (5.00-6.15)	5.45 (3.47-6.98)	5.32 (3.60-5.73)
BSC	5.4 (5.40-6.15)	5.4 (5.00-6.15)	6.3 (5.40-7.20)	6.3 (5.55-7.20)
HR (beats/min)	95 ± 18	87 ± 6	118 ± 31	134 ± 15
SBP (mmHg)	155 ± 28	152 ± 36	155 ± 28	155 ± 33
DBP (mmHg)	91 ± 10	95 ± 18	88 ± 15	93 ± 19
MAP (mmHg)	127 ± 23	111 ± 19	110 ± 18	117 ± 19

The values of BW and BCS are presented as median (interquartile range). The values of HR, PR, SBP, DBP, and MAP are presented as mean ± SD. *BCS* body condition score, *BW* body weight, *HR* heart rate, *PR* pulse rate, *SBP* systolic blood pressure, *DBP* diastolic blood pressure, *MAP* mean arterial blood pressure

### Short-term (4 weeks) melatonin supplement did not affect blood profiles of MMVD dogs

There were no statistically significantly differences in hematology and blood chemistry profiles between pre- and post-treatment (4 weeks) in placebo and melatonin administration. This study suggests that short-term melatonin supplementation (2 mg/kg) for 4 weeks does not affect blood profiles in MMVD dogs (Table 2).

**Table 2:** Complete blood count and biochemistry profiles of myxomatous mitral valve degenerative dogs pre- and post-treatment in the control and treatment groups

Parameter	Control group (*n* = 11)		Treatment group (*n* = 18)		Normal range [28]
	Pre-treatment	Post-treatment	Pre-treatment	Post-treatment	
Packed cell volume (%)	46.82 ± 3.25	47.50 ± 6.80	46.38 ± 7.43	47.50 ± 6.60	35-57
Hemoglobin (g/dI)	15.28 ± 1.36	15.44 ± 2.23	15.26 ± 2.34	15.63 ± 2.19	11.9-18.1
RBC count (× 10^6^ cells/μl)	6.28 ± 0.71	6.35 ± 1.01	6.59 ± 1.01	6.68 ± 0.86	4.95-7.87
MCV (fl)	73.99 ± 4.61	74.18 ± 3.21	70.36 ± 3.36	70.02 ± 3.99	60-77
MCHC (g/dI)	32.62 ± 0.88	32.90 ± 0.88	32.98 ± 0.73	32.91 ± 0.97	32.0-36.3
WBC count (10^3^ cells/μl)	10.62 ± 3.47	9.18 ± 2.96	10.41 ± 2.22	10.13 ± 2.67	5-14.1
**Segmented neutrophil (× 10**^**3**^ **cells/μl)**	6.66 ± 2.19	5.64 ± 1.88	6.81 ± 1.89	6.96 ± 2.04	2.9-12
Lymphocyte (10^3^ cells/μl)	2.75 ± 2.64	2.6 ± 2.26	2.12 ± 1.07	1.82 ± 0.70	0.4-2.9
Monocyte (10^3^ cells/μl)	0.63 ± 0.43	0.42 ± 0.28	0.81 ± 0.48	0.75 ± 0.68	0.1-1.4
Eosinophil (10^3^ cells/μl)	0.75 ± 0.71	0.51 ± 0.36	0.71 ± 0.50	0.60 ± 0.44	0-1.3
Basophil (10^3^ cells/μl)	0.02 ± 0.04	0.01 ± 0.02	0.01 ± 0.01	0.01 ± 0.01	0-0.14
Platelet count (10^3^ cells/μl)	349.67 ± 120.6	354.00 ± 110.7	395.53 ± 135.9	367.17 ± 122.6	211-621
BUN (mg/dI)	18.57 ± 6.63	23.58 ± 15.83	23.58 ± 18.85	26.28 ± 10.71	8-28
Creatinine (mg/dI)	1.07 ± 0.36	1.05 ± 0.23	1.18 ± 0.30	1.30 ± 0.31	0.5-1.7
ALT (U/L)	65.45 ± 41.66	61.11 ± 39.74	58.80 ± 31.33	59.53 ± 32.21	10-109
ALP (U/L)	88.00 ± 98.82	85.88 ± 84.81	86.38 ± 48.89	84.00 ± 47.44	1-114
Total protein (g/dI)	7.85 ± 0.84	7.73 ± 0.74	7.83 ± 0.85	7.48 ± 0.94	5.4-7.5
Albumin (g/dI)	3.13 ± 0.36	3.04 ± 0.29	3.23 ± 0.24	3.18 ± 0.23	2.3-3.1

Values are presented as mean ± SD. *RBC* red blood cell, *MCV* mean corpuscular volume, *MCHC* mean corpuscular hemoglobin concentration, *WBC* white blood cell, *BUN* blood urea nitrogen, *ALT* alanine aminotransferase, *ALP* alkaline phosphatase

### Short-term (4 weeks) melatonin supplement did not improve cardiac structure or function in MMVD dogs

Regarding normalization of LA diameter and LA:AO ratio, MMVD dogs in both the control and treatment groups had an increase in normalization of the LA diameter compared with a 95% prediction interval as well as an increase in the LA:AO ratio compared to pre-treatment as referenced in ACVIM guidelines [29, 30]. However, at post-treatment, these parameters were not significantly different when compared to the pre-treatment values for the same group (Tables 3 and 4).

**Table 3:** Normalization with bodyweight of Motion (M)-mode parameters of myxomatous mitral valve degenerative dogs pre- and post-treatment in the control and treatment groups

M-mode Parameter	Control group (*n* = 11)		Treatment group (*n* = 18)		95% prediction interval (cm) [31]
	Pre-treatment	Post-treatment	Pre-treatment	Post-treatment	
LA diameter (cm)	1.11 ± 0.05	1.06 ± 0.14	1.25 ± 0.21	1.19 ± 0.19	0.59-0.97
AO diameter (cm)	0.74 ± 0.10	0.76 ± 0.09	0.75 ± 0.08	0.73 ± 0.10	0.63-0.96
IVSDN (cm)	0.48 ± 0.1	0.45 ± 0.08	0.41 ± 0.09	0.42 ± 0.1	0.29-0.59
LVIDdN (cm)	1.53 ± 0.28	1.60 ± 0.25	1.69 ± 0.3	1.65 ± 0.32	1.27-1.85
LVPWdN (cm)	0.49 ± 0.14	0.47 ± 0.07	0.44 ± 0.09	0.43 ± 0.07	0.29-0.60
IVSsN (cm)	0.64 ± 0.12	0.68 ± 0.16	0.65 ± 0.11	0.64 ± 0.13	0.43-0.79
LVIDsN (cm)	0.85 ± 0.21	0.79 ± 0.15	0.81 ± 0.16	0.80 ± 0.23	0.71-1.26
LVPWsN (cm)	0.69 ± 0.09	0.75 ± 0.11	0.75 ± 0.13	0.70 ± 0.12	0.48-0.87

Values are presented as mean ± SD. *LA* left atrium, *AO* Aortic root, *IVSdN* normalized interventricular septum thickness end-diastole, *LVIDdN* normalized left ventricular internal diameter end-diastole, *LVPWdN* normalized left ventricular posterior wall end diastole, *IVSsN* normalized interventricular septum thickness end systole, *LVIDsN* normalized left ventricular internal diameter end systole, *LVPWsN* normalized left ventricular posterior wall end-systole

**Table 4:** Cardiac function and left atrial size from Motion (M)-mode parameters of myxomatous mitral valve degenerative dogs pre- and post-treatment in the control and treatment groups

Parameter	Control group (*n* = 11)		Treatment group (*n* = 18)	
	Pre-treatment	Post-treatment	Pre-treatment	Post-treatment
LA:AO ratio	1.61 ± 0.16	1.64 ± 0.16	1.79 ± 0.28	1.64 ± 0.16
EDV (mL)	24.94 ± 11.33	28.47 ± 13.55	28.80 ± 12.60	28.47 ± 13.55
EF (%)	71.83 ± 17.29	80.94 ± 8.57	80.54 ± 10.71	80.94 ± 8.57
ESV (mL)	6.96 ± 4.41	5.67 ± 4.25	5.07 ± 3.63	5.67 ± 4.25
FS (%)	41.82 ± 13.27	44.13 ± 18.43	49.89 ± 8.82	48.68 ± 10.26
SV (mL)	17.98 ± 8.81	20.73 ± 12.48	22.41 ± 12.25	22.80 ± 10.98

Values are presented as mean ± SD. *LA:AO* left atrial on aortic, *EDV* end-diastolic volume, *%EF* % ejection fraction, *ESV* end-systolic volume, *%FS* % fractional shortening, *SV* stroke volume

Other M-mode parameters pre-treatment were within standard limits in both the control and treatment groups (Tables 3 and 4). Whether a dog received melatonin or placebo, the other M-mode parameters and cardiac function parameters investigated by echocardiography were not significantly different from pre-treatment values within the same group. This study suggests that short-term melatonin supplementation (2 mg/kg) for 4 weeks does not exert a cardioprotective effect as evidenced by M-mode echocardiographic parameters in post-treatment compared to pre-treatment in MMVD dogs (Tables 3 and 4).

### Short-term melatonin supplementation for 4 weeks reduced plasma MDA in MMVD dogs

For MDA determination, after we identified the statistical outliers of MDA levels, we excluded one dog with MMVD stage C in the treatment group. The total number of dogs in the control group was eleven, whereas the number of dogs in the treatment group was reduced to seventeen.

**Fig. 1:**
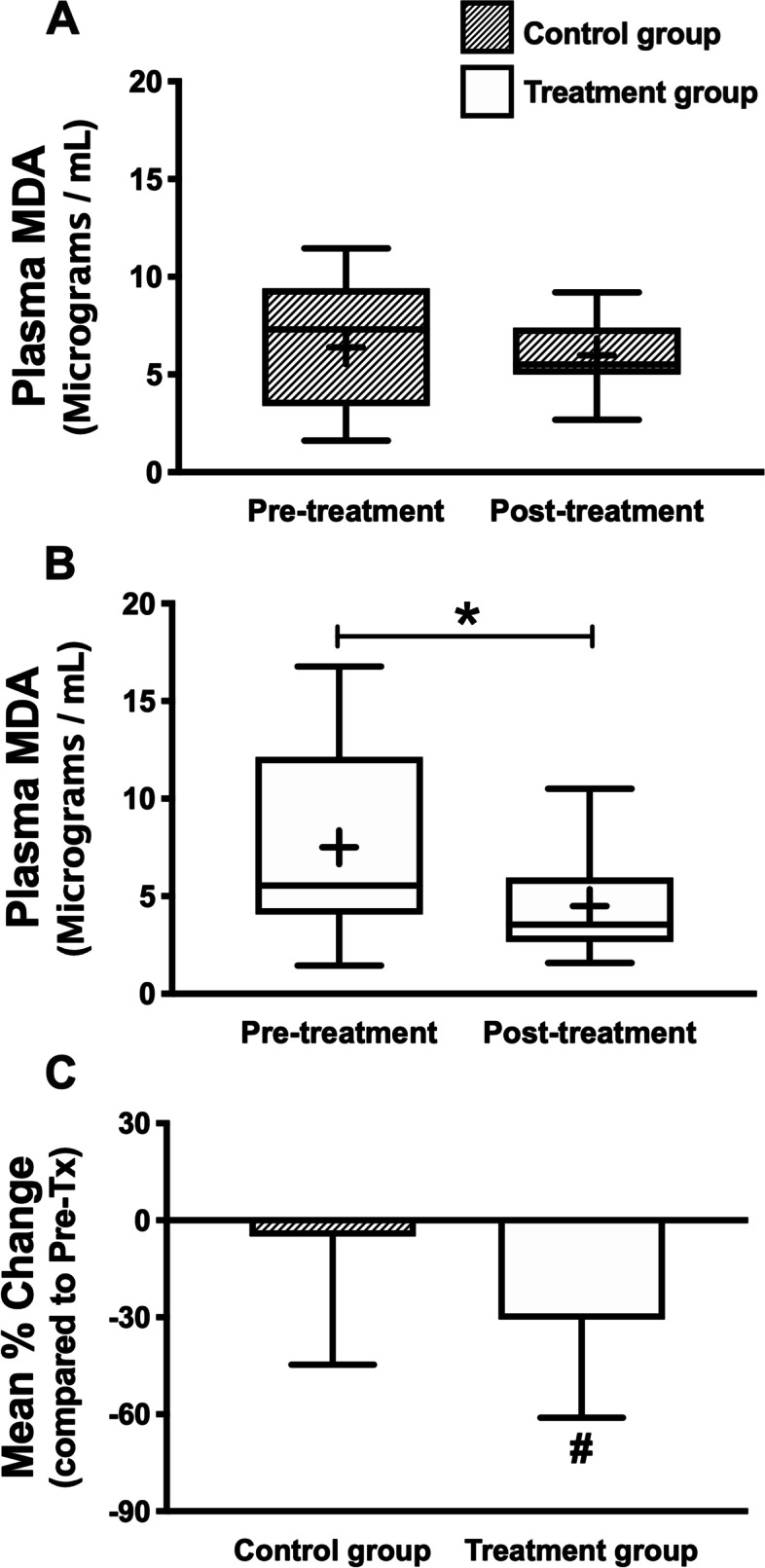
Plasma MDA level pre-and post-treatment in the control (**A**) and the treatment group (**B**) and the mean percentage (%) change of plasma MDA level in the control and treatment group (**C**). The plasma MDA level was not different between pre-and post-treatment in the control group (**A**). However, melatonin supplementation in the treatment group exerted a beneficial effect on MDA level, increasing attenuation of plasma MDA post-treatment compared to pre-treatment (**B**). Melatonin also increased the mean % change of plasma MDA level in the experimental group compared to the control group (**C**). (* *P* < 0.05 vs. pre-treated in the same group, ^#^ *P* < 0.05 vs. control group). MDA = malondialdehyde, Pre-Tx = pre-treatment

The MDA level detected by the HPLC system and the mean plasma MDA concentrations were not statistically significantly different between pre-treatment and post-treatment in the control group (Fig. 1A). However, in the melatonin treatment group, the mean plasma MDA concentration significantly decreased from 7.51 ± 4.62 μg/mL pre-treatment to 4.50 ± 2.60 μg/mL after treatment for 4 weeks (*p* = 0.038) (Fig. 1B). Moreover, the decrease in the proportion of MDA in the treatment group was higher than the control group as indicated by the larger mean % change of the treatment group than that of the control group (*p* < 0.05) (Fig. 1C). This result suggests that melatonin supplementation reduces MDA’s proportion better than the control group.

The plasma MDA levels of the pre-and post-treatment varying with the staging of MMVD were evaluated and are presented in Table 5. In the control group, the plasma MDA level was not significantly different from the MMVD stage B2 and the MMVD stage C dogs at each timepoint (pre-and post-treatment) and there was no significantly different between pre-treatment and post-treatment within stages. In addition, the plasma MDA level in the treatment group demonstrated not significantly different between MMVD stage B2 and MMVD stage C at each timepoint (pre-and post-treatment). Interestingly, the present study found that the plasma MDA level decreased in both MMVD stage B2 and MMVD stage C dogs compared to pre-treatment. However, a statistically significant difference between pre-and post-treatment was found only in the treated dogs with MMVD stage C (Table 5). This suggests that short-term melatonin supplementation (2 mg/kg) for 4 weeks could attenuate plasma MDA in some MMVD dogs.

**Table 5:** Plasma malondialdehyde concentration pre- and post-treatment in the control and treatment groups varying by stage in myxomatous mitral valve degenerative dogs

Control group (*n* = 11)	Plasma MDA (μg/mL)	
	**Pre-treatment**	**Post-treatment**
MMVD stage B2 (*n* = 5)	4.59 ± 2.87	4.70 ± 1.26
MMVD stage C (*n* = 6)	7.90 ± 3.30	7.06 ± 1.76
Treatment group (*n* = 17)	**Plasma MDA (μg/mL)**	
	**Pre-treatment**	**Post-treatment**
MMVD stage B2 (*n* = 6)	7.84 ± 5.71	3.48 ± 2.01
MMVD stage C (*n* = 11)	7.33 ± 4.22	5.05 ± 2.81 *

Values are presented as mean ± SD. * *P*-value < 0.05 vs. Pre-treatment in the same group; *MDA* malondialdehyde, *MMVD* myxomatous mitral valve degeneration

## Discussion

The present study demonstrated the effects of short-term (4 weeks) melatonin administration in cardiac remodeling of MMVD dogs with no adjustment of standard prescriptions. The major findings of this study are as follows. First, melatonin supplement (2 mg/kg) once a day orally for 4 weeks did not affect vital signs, hematology, or blood chemistry profiles in cardiac remodeling MMVD dogs. Second, although melatonin supplementation for 4 weeks attenuated plasma MDA levels, melatonin supplements did not have any effects, either beneficial or adverse, on cardiac structural changes or cardiac function based on investigation by echocardiography in the MMVD dogs.

In veterinary medicine, melatonin is used to treat skin diseases such as alopecia-X in dogs. The dosage is 1 mg/kg orally twice a day or between 2 and 3 mg/kg orally once daily for from 1 week to 3 months [32–34]. Interestingly, a previous study in dogs using melatonin 1 mg/kg (peroral) twice a day reported that melatonin therapy does not affect IL-2 or IFN-γ expression in healthy dogs [35]. Another study in Pomeranian dogs with hair cycle arrest (alopecia X) using melatonin 1 mg/kg (peroral) twice a day reported that melatonin supplement has beneficial effects on increasing hair growth and decreasing epidermal pigmentation [34]. The effect of melatonin on the heart or cardiac structure and function in dogs, however, has never been investigated.

Additionally, cardiac remodeling responds to many pathological factors [36–38]. Volume overload can cause cardiac wall stress and can alter myocardial oxygen demand, increasing oxidative stress levels [38, 39]. Moreover, previous studies in dogs have demonstrated the relationship between cardiac disease-induced cardiac remodeling and increased oxidative stress [40–42]. The association between oxidative injuries and heart diseases has been widely studied. The reduction of oxidative stress is one of the goals of improving therapeutic strategies for heart disease in animal models [7, 40, 41, 43]. Theoretically, finding drugs that reduce oxidative stress levels would improve cardiac function at the cellular level and improve whole-organ function [44].

This study found that melatonin (2 mg/kg orally, once a day) did not affect vital signs (HR and blood pressure), body weight, body condition score, or blood profiles after treatment for 4 weeks. However, this result is not consistent with a previous clinical study in humans with metabolic syndrome which reported that using melatonin (5 mg/kg for 8 weeks) could lower blood pressure and body mass index [45]. A previous study of rats fed a high-fat diet reported that melatonin supplementation (30 mg/kg for 3 weeks) could reduce body weight gain and improve metabolic impairment [46], while another study in rats fed a high-fat diet reported that chronic continuous melatonin administration in drinking water (10 mg/kg for 8 weeks) could reduce weight gain and total serum cholesterol levels [47]. These discrepancies might be due to the specific experimental models, the dosage, and/or the duration of melatonin supplementation. Further studies in dogs with MMVD need to be conducted.

Moreover, this study demonstrated that melatonin supplementation at dosage 2 mg/kg for 4 weeks does not exert a cardioprotective effect indicated by echocardiograms. A limitation of this study was the relatively low dosage and short duration of melatonin administration compared with other studies [18, 25, 48, 49]. This could be why melatonin did not improve clinical outcomes as measured by vital signs as well as cardiac structure and function indicated by M-mode echocardiographic parameters in the MMVD dogs. Additional studies with larger numbers of dogs are needed to determine the proper dosage and administration duration of melatonin to improve clinical outcomes in MMVD dogs.

In addition, the present study using melatonin 2 mg/kg found that it could decrease oxidative stress but that it did not exhibit obvious effects on cardiac structure or function. On the other hand, previous studies in laboratory animal models have reported that melatonin exhibits a cardioprotective effect and that it can improve cardiac function at variety dosages, e.g., 20-50 mg/kg, in mice with myocardial infarction or cardiac dysfunction [48, 50, 51] and 7-30 mg/kg in rats with ischemia and reperfusion injury [52–54]. Although melatonin at 2 mg/kg once a day is safe in dogs when given for a month, this dosage and duration of treatment might not be sufficient to exhibit a cardioprotective effect in clinical practice with the exception of attenuated oxidative stress in dogs with MMVD. Additionally, the standard medical therapy used in each stage of MMVD in this study might have had an effect on the clinical outcomes.

Moreover, this study had compared plasma MDA levels pre-treatment and post-treatment in each group and found that melatonin administration can attenuate plasma MDA levels in the treatment group better than the control group. In addition, the study found that some of the dogs with MMVD stage C had higher MDA levels than those some of the dogs with MMVD stage B, whereas some dogs with MMVD stage C had a level of MDA lower than or equal to those with MMVD B2. On the other hand, we also found that some dogs with MMVD B2 had higher MDA levels than dogs with MMVD stage C. The results of this clinical study suggest that not all dogs with MMVD stage C have higher MDA than dogs with MMVD stage B2, and are consistent with a previous study in MMVD dogs which reported that oxidative stress markers (MDA and oxLDL) are not associated with the clinical stage of MMVD [55]. Although there have been reports indicating that oxidative stress is not related to the clinical stage of MMVD in dogs, those findings remain controversial [8, 56–58]. The variability of MDA in this study might be due to clinical differences between the groups which could not be controlled for in the clinical study, e.g., food, physiological stress, and other subclinical health problems which cannot be detected by a general physical examination and standard laboratory screening tests. A higher number of MMVD dogs in each stage is needed to confirm the relationship between oxidative stress and the staging of MMVD in dogs.

Moreover, although the treatment group had more dogs with MMVD stage C than the control group, the results showed that melatonin supplementation in both MMVD stage B2 and stage C dogs was associated with a downward trend in plasma MDA levels between pre-treatment and post-treatment. The change in the mean MDA values from pre- to post-treatment in MMVD stage B2 dogs in the treatment group was greater than that of the MMVD stage C dogs from the treatment group, but the pre- to post- level difference reached statistical significance only in MMVD stage C dogs. This result might be due to the small sample size and high variation of MDA level in MMVD stage B2 dogs in the treatment group. Further clinical study with a larger population is needed to evaluate this finding.

This finding does, however, appear to support the hypothesis that melatonin has a cardioprotective effect in cardiac physiology based on its demonstrated antioxidant properties. The present study is in concordance with many previous studies which have reported the cardioprotective effect of melatonin on heart failure in human and animal models as well as in in vitro studies [48, 54, 59, 60]. The predominant antioxidant mechanisms of melatonin include direct free radical scavenging of oxygen-based free radicals and related free radical species, stimulation of antioxidative enzymes, increased mitochondrial efficiency of oxidative phosphorylation, reduced electron leakage and augmentation of the efficiency of other antioxidants [61].

Regarding melatonin’s physiological role, melatonin can directly reduce nitric oxide (NO) generation within mitochondria as well as increase mitochondrial respiration, ATP production and electron transportation [62]. Moreover, a study by Liu and colleagues on the effect of melatonin on a falling heart found that it may potentially play a role in the upregulation of antioxidative hormone levels, including superoxide dismutase (SOD) and glutathione peroxidase (GPx), by increasing both SOD2 and GPx mRNA levels [48]. Plasma MDA is also a cardiovascular-related non-specific circulating oxidative biomarker that can be produced by any cell membrane throughout the body [9]. Thus, further large-scale study of melatonin administration and cardiac-specific biomarkers such as cardiac tissue MDA, cardiac troponin (cTn), B-type natriuretic peptide (BNP), or N-terminal pro B-type natriuretic peptide (NT-proBNP) levels could be helpful in clinical practice.

Moreover, studies with high dosages and long periods of melatonin administration with the same staging of MMVD but which are breed-specific, have a larger sample size and include more echocardiographic parameters, e.g., Doppler, global systolic and diastolic function measurement, are needed to test the hypotheses generated in this study. In addition, further studies of the feasibility and effectiveness of melatonin supplements in MMVD dogs are needed to fill in this gap in knowledge.

## Conclusions

A short-term melatonin supplement (2 mg/kg) once a day orally for 4 weeks could attenuate the plasma MDA in cardiac remodeling MMVD dogs. However, this supplement does not have any beneficial or adverse effects on echocardiographic parameters, blood profiles, or vital signs in cardiac remodeling MMVD dogs. Although MDA variation was found in both MMVD stage B2 and stage C, melatonin supplementation demonstrated a downward trend in plasma MDA levels between pre-treatment and post-treatment in both stages. Thus, short-term melatonin oral supplements, especially as an adjunct to the standard treatment protocol, could reduce oxidative stress levels but would not exert a beneficial effect on cardiac structural changes or cardiac function in MMVD dogs. Further study is needed to test the hypotheses generated in this study.

## Methods

### Animal model and research protocol

Twenty-nine small breed dogs (Poodle, Chihuahua, Shih Tzu, and Pomeranian) with mitral valve degeneration (MMVD stages B2 and C) aged 7 to 16 years and weighing between 3 to 10 kg were enrolled in the study. MMVD staging was categorized in accordance with a previous study and ACVIM guidelines [29, 30]. Stage B2 refers to asymptomatic MMVD dogs with cardiac enlargement as seen in radiographs and/or echocardiographs [30]. Stage C refers to MMVD dogs which exhibit current or past clinical signs of heart failure that is under control with standard medical treatment [30].

All the dogs with MMVD stage B2 and stage C were in a stable condition and had continuously received the standard medical therapy with no change in the therapeutic dosages. The dogs with MMVD stage B2 were given an inotropic drug (Pimobendan, 0.25-0.3 mg/kg twice a day) and an angiotensin-converting enzyme inhibitor (Ramipril, 0.125-0.25 mg/kg once a day). The dogs with MMVD stage C received the same drugs as the MMVD stage B2 dogs plus diuretic drugs (Furosemide, 1-3 mg/kg twice a day and Spironolactone, 2 mg/kg once a day). None of the dogs in the study received any antioxidant supplementation, e.g., omega-3, omega-6, vitamin E, or vitamin C. All MMVD dogs who visited the small animal hospital were given general screening tests including history taking, physical examination, thoracic radiography, and blood collection. Dogs which were found to have cardiac disorders other than MMVD during cardiovascular examination were excluded from the study. Dogs were also excluded if they had any abnormalities in hematology and/or blood chemistry profiles indicative of systemic diseases, inflammation/infection, metabolic diseases, kidney diseases, or neoplasm.

All MMVD dogs were prescribed the same standard therapy and were randomly divided into two groups: a control (placebo) group (*n* = 11) consisting of MMVD stage B2 (*n* = 5) and MMVD stage C (*n* = 6) and a treatment group (*n* = 18) consisting of MMVD stage B2 (*n* = 6) and MMVD stage C (*n* = 12). The control group was comprised of 5 females and 6 males, whereas the treatment group included 7 females and 11 males. Melatonin (Circadin® 2 mg/kg, RAD Neurim Pharmaceuticals, Berkshire, UK) or a placebo (corn starch tablet) were prescribed once a day at evening time (PM) for 4 weeks in the treatment group and control group, respectively. A general physical examination was conducted every 7 days and a record of the amount of melatonin used was maintained. Blood collection to determine blood profile and plasma oxidative stress (MDA level) and blood pressure as well as echocardiography were performed on Day 0 and on Day 28 of drug administration. Plasma was kept at − 80 °C for MDA analysis [12] which was conducted at the end of the study. All owners were informed of the procedures and signed a consent form before the start of the study. All experiments were approved by the Animal Care and Use Committee, Faculty of Veterinary Medicine, Chiang Mai University (Ethical number: S17/2562).

### Physical examination

The health status of all the dogs was evaluated by standard physical examination (inspection, palpation, percussion, and auscultation), including general appearance, vital signs such as mucous membrane color, capillary refill time, temperature, heart rate, pulse rate, and respiratory rate, non-invasive blood pressure measurement (Oscillometric method), palpation and auscultation. Body condition was evaluated by the same principal investigator using the nine-scale body condition score (BCS) system [63].

### Blood pressure evaluation

Blood pressure (BP) measurements were performed with an oscillometer (CARESCAPE™ V100, GE healthcare, Milwaukee, WI, USA) and evaluated following American College of Veterinary Internal Medicine (ACVIM) consensus statement guidelines for the identification, evaluation, and management of systemic hypertension in dogs and cats [64]. Either the left or the right forelimb circumference was measured using a cuff corresponding to 40% of the limb’s circumference. Systolic blood pressure (SBP), diastolic blood pressure (DBP), and mean arterial pressure (MAP) were also recorded. The average of five consecutive BP measurements was used for statistical analysis. Dogs with systemic hypertension were excluded from the study.

### Blood collection for hematology, serum biochemistry profiles

Five milliliter fasting blood samples were collected by venipuncture from the cephalic or the saphenous vein at the beginning (day 0) and the end of the study (day 28). Blood samples were divided into two portions. The first portion was placed in a potassium ethylene diamine tetra-acetic acid (EDTA) tube for the hematology profile. The second portion was placed in a lithium-heparin tube and centrifuged at 3000 rpm for 15 min for blood chemistry profile analysis and the MDA test. Plasma for the MDA test was stored at − 80 °C until analyzed.

### Measuring levels of plasma malondialdehyde (plasma MDA)

After we identified the MDA level statistical outliers, we excluded one dog in the treatment group with MMVD stage C. The total number of dogs in the control group was eleven, whereas the number of dogs in the treatment group was reduced to seventeen.

Plasma MDA levels were measured using a high-performance liquid chromatography (HPLC) system (Thermo Scientific, Bangkok, Thailand) [43, 65]. Plasma was mixed with a 0.44 M H_3_PO_4_ and 0.6% thiobarbituric acid (TBA) solution, resulting in the generation of pink-colored products called thiobarbituric acid reactive substances (TBARS). Plasma TBARS concentrations were determined directly from a standard curve and reported as an MDA equivalent concentration by an HPLC-based assay [66].

### Echocardiographic parameter determination

Cardiac walls, cardiac chambers, and cardiac function were evaluated by an experienced veterinarian using echocardiography (Philip® CX50, Bothell, USA) in 2D and 2D-guided M-mode with 3.8-6 MHz transducers. Dogs were shaved between the 4th and 6th right intercostal spaces from the costochondral junction to the sternum. The experienced veterinarian who performed the echocardiography throughout the study did not know the dog’s data or group while doing the echocardiography (randomized and blinded techniques). LV wall thickness, LV dimension, and LV function were evaluated by M-mode echocardiography in the right para-sternal short axis (at the base of the heart and the level of the papillary muscles) [67]. The left ventricular free wall thickness during diastole and systole (LVPWd, LVPWs), left ventricular internal dimensions in diastole and systole (LVIDd, LVIDs) as well as interventricular septum thickness in diastole and systole (IVSd, IVSs) were measured. All averaged M-mode chamber measurements were normalized by body weight using the Cornell allometric scale for dogs [31]. M-mode echocardiography parameters were used to calculate the percentage of fractional shortening (%FS), and the Teicholz formula was used to calculate the percentage ejection fraction (%EF), the end-systolic volume (ESV), and the end-diastolic volume (EDV) which was accomplished automatically by the echocardiographic equipment software. The right parasternal short-axis view was used to measure the left atrial dimension (LA) and aortic dimension (AO) in early diastole. AO was measured at the level of the aortic valve, whereas LA was measured in the left atrial dimension. Then the left atrial to aortic root ratio (LA:AO ratio) was calculated using the Swedish method. Three consecutive beats of cardiac cycles were measured, and the average values were used for all echocardiographic parameters.

### Statistical analysis

All continuous variables were tested for normal distribution using the Shapiro-Wilk test. A *P*-value greater than 0.05, the standard for statistical significance, was used for assessment of the normality of the data. Normally distributed data are presented as mean ± SD, and non-normally distributed data are shown as median (interquartile range). For each group, data of blood pressure, hematology, and serum chemistry profiles pre- and post-treatment were compared using the paired T-test. If the data did not meet the normality assumption, the Wilcoxon Signed-Rank test was used. In addition, mean levels of MDA before and after treatment were compared within each group using the paired T-test. The percentage change of MDA level before and after treatment in each group was calculated and the values were compared between the control and treatment groups using the general linear model to account for the unequal sample size.

## Data Availability

The datasets used during the current study are available from the corresponding author upon reasonable request.

## Abbreviations

2D: Two dimensional
ACVIM: American College of Veterinary Internal Medicine
AO: Aortic dimension
ALT: Alanine aminotransferase
ALP: Alkaline phosphatase
BCS: Body condition score
BNP: B-type natriuretic peptide
BP: Blood pressure
BUN: Blood urea nitrogen
cTn: Cardiac troponin
DBP: Diastolic blood pressure
EDTA: Potassium ethylene diamine tetra-acetic acid
EDV: End-diastolic volume
EF: Ejection fraction
eNOS: Endothelial nitric oxide synthase
ESV: End-systolic volume
FS: Fractional shortening
FOX: Ferrous oxidation of xylenol orange
GPx: Glutathione peroxidase
GSH: Glutathione
HF: Heart failure
HPLC: High-performance liquid chromatography
IL-2: Interleukin-2
IFN-γ: Interferon gamma
IVSd: Interventricular septum thickness in diastole
IVSdN: Normalized interventricular septum thickness end-diastole
IVSs: Interventricular septum thickness in systole
IVSsN: Normalized interventricular septum thickness end-systole
LA: Left atrium dimension
LA:AO ratio: Left atrial to aortic root ratio
LVEF: Left ventricular ejection fraction
LVIDd: Left ventricular internal dimensions in diastole
LVIDdN: Normalized left ventricular internal diameter end-diastole
LVIDs: left ventricular internal dimensions in systole
LVIDsN: Normalized left ventricular internal diameter end systole
LVPWd: Left ventricular free wall thickness during diastole
LVPWdN: Normalized left ventricular posterior wall end diastole
LVPWs: Left ventricular free wall thickness during systole
LVPWsN: Normalized left ventricular posterior wall end-systole
MAP: Mean arterial pressure
MCHC: Mean corpuscular hemoglobin concentration
MCV: Mean corpuscular volume
MDA: Malondialdehyde
M-mode: Manual mode
MMVD: Myxomatous mitral valve degenerative
NO: Nitric oxide
NT-proBNP: N-terminal pro B-type natriuretic peptide
RBC: Red blood cell
ROS: Reactive oxygen species
SBP: Systolic blood pressure
SCN: Suprachiasmatic nucleus
SOD: Superoxide dismutase
SV: Stroke volume
TBARS: Thiobarbituric acid reactive substances
TNF-α: Tumor necrosis factor-α
WBC: White blood cell
